# SARS COV-2 and other viral etiology as a possible clue for the olfactory dilemma

**DOI:** 10.1186/s43163-022-00251-9

**Published:** 2022-05-21

**Authors:** Ossama I. Mansour, Mohamed Shehata Taha, Mohammad Salah Mahmoud, Waleed Farag Ezzat, Anas Askoura, Mohamed Farouk Allam, Samia Abdo Girgis, Azza Omran, Sara Hassan Agwa, Mohamed Naguib Mohamed

**Affiliations:** 1 Department of Otolaryngology, Faculty of Medicine, Ain Shams University, 38 Abbassia, Next to the Al-Nour Mosque, Cairo, Egypt; 2 Department of Family Medicine, Faculty of Medicine, Ain Shams University, 38 Abbassia, Next to the Al-Nour Mosque, Cairo, Egypt; 3 Department of Clinical Pathology, Faculty of Medicine, Ain Shams University, 38 Abbassia, Next to the Al-Nour Mosque, Cairo, Egypt; 4 Department of Clinical Pathology, El-Mataria Teaching Hospital, Cairo, Egypt; 5 Clinical Pathology, Molecular Genomic Unit, MASRI, Ain Shams University, 38 Abbassia, Next to the Al-Nour Mosque, Cairo, Egypt

**Keywords:** Anosmia, SARS-CoV-2, Incidence, Recovery, Seroconversion, COVID-19

## Abstract

**Background:**

Post-viral anosmia is responsible for more than 40% of cases of anosmia. Anosmia has been a neglected symptom in the primary healthcare setting until the emergence of the SARS-CoV-2 pandemic. The spread of SARS-CoV-2 infection highlighted new atypical symptoms of the disease, including anosmia, which has become one of the diagnostic symptoms of the disease, and epidemiological concern. We aimed to detect the incidence of SARS-CoV-2 infection within patients presented with anosmia and to test for other respiratory viruses in the negative COVID-19 patients. We also detected the recovery of anosmia and IgM/IgG against COVID-19. We prospectively included 60 outpatients with the major complaint of anosmia. Nasopharyngeal swabs were done for SARS-CoV-2 real-time PCR, and if negative, PCR to other respiratory pathogens was tested. After one month, we inquired about the recovery of smell loss together with testing for antibodies against SARS-CoV-2.

**Results:**

Sixty patients were enrolled in the study. Forty-six patients (76.7%) were SARS-CoV-2 PCR positive and 14 (23.3%) were negative. Rhinovirus was the commonest isolated pathogen in the negative cases (5/14). Complete recovery of anosmia occurred in 34 patients (56.7%), while partial recovery in 24 (40.0%), and no recovery in 2 patients (3.3%). The median time to complete recovery was 10 days. 28.3% (13/46) of the patients showed negative antibody response for both IgG and IgM.

**Conclusions:**

Sudden-onset anosmia is a symptom that is highly predictive of being COVID-19-infected. While recovery is expected within 2 weeks, some patients have no antibodies against SARS-CoV-2.

## Background

Post-viral anosmia is responsible for more than 40% of cases of anosmia, especially in adults. There are more than 200 different types of viruses that produce common cold and upper respiratory tract infection, among them the coronaviruses, which are first characterized in the 1960s and are accounting for 10–15% of cases [1], Coronaviruses are a large family that results in a variety of diseases ranging from a common cold to massive public health concerns [2].

As of June 22th, 2021, more than 494,587,638 people worldwide have severe acute respiratory syndrome coronavirus 2 (SARS-CoV-2) with 6,170,283 deaths [3]. Asian clinical studies reported that the common symptoms consist of fever, cough, difficult breathing, expectoration, muscle aches, joint pains, headache, diarrhea, and sore throat [4, 5]. The spread of the COVID-19 infection outside Asia has emphasized new atypical symptoms of the disease as many COVID-19-infected patients presented with anosmia and ageusia without fever, nasal congestion, or rhinorrhea. The British Rhinology Society and the European Rhinology Society released recommendations that loss of smell can be the presenting symptom or even the only symptom of contracting COVID-19 infection [6, 7].

Further studies were published regarding olfactory dysfunctions in COVID-19 patients reporting that its prevalence ranges from 19.4 to 98.33% [8]. A huge peer-reviewed report from UK and USA indicates that two-thirds of positive COVID-19 self-reported cases complained of loss of smell or taste [9]. Another systematic review states that 30% to 80% of confirmed COVID-19 patients complain of loss of smell or taste [10].

The manifestation of anosmia as a post-viral sequela is not a novel symptom in the field of otolaryngology as numerous viruses might cause post-viral olfactory dysfunction, so we aimed to detect the incidence of SARS-CoV-2 infection within patients presented with a major complaint of anosmia and to test for other upper respiratory viruses in the negative COVID-19-infected patients. We also detected the recovery of anosmia and the presence of IgM/IgG of COVID-19 after 1 month from the presentation.

## Methods

After the study has been approved by the Research Ethics Committee of Ain Shams University Faculty of Medicine with reference number FMASU P45a/2020 on July 11th, 2020, patients were invited to participate.

Sixty patients presenting to the Triage and/or Otolaryngology clinics in Ain Shams University Hospitals with anosmia as the main complaint; underwent thorough history taking, clinical examination, subjective assessment of anosmia and rt-PCR to SARS-COV2 by using VIASURE SARS-CoV-2 Real-Time PCR kits (CerTest Biotec®, S.L., Spain); and if proven negative to COVID-19, PCR to other respiratory pathogens was tested using multiplex reverse-transcriptase real-time polymerase chain reactions (rRT-PCR) from the FTD® Respiratory Pathogens 33 multiplex tests (Fast Track Diagnostic FTD®, Luxembourg). This kit is used for detection of the following respiratory viruses: influenza A, influenza A subtype A (H1N1), influenza B, and influenza C; parainfluenza viruses 1, 2, 3, and 4; coronaviruses NL63, 229E, OC43, and HKU1; human metapneumoviruses A and B; rhinovirus; respiratory syncytial viruses A and B; adenovirus; enterovirus; parechovirus; human bocavirus. COVID-19-infected patients were managed according to the standard protocol of ***** University Hospitals. Follow-up after 1 month for the outcome of recovery of the smell sense (subjective assessment) and seroconversion by finger-prick rapid test for IgM/IgG of COVID-19 using Artron® COVID-19 IgG/IgM Antibody Test Kit (Artron Laboratories Inc., Canada), which is a test kit used for the qualitative detection of IgM and IgG against SARS-CoV-2 in human whole blood or serum with a combined sensitivity for both IgM and IgG of 91.40%, and a specificity of 97.88%

The following inclusion criteria have been considered: adults more than 18 years old and presenting with acute-onset anosmia.

The following exclusion criteria have been considered: patients with a history of smell loss before the epidemic; patients with a known history of chronic rhinosinusitis or sinonasal surgery; and patients with neurodegenerative diseases.

### Statistical methods

Categorical variables are presented as numbers and percentages and intergroup differences as compared using the Pearson chi-squared test or Fisher’s exact test. Normally distributed numerical data are presented as mean ± standard deviation and differences are compared using the independent-samples *t* test. Skewed numerical data are presented as median and interquartile range and differences are compared with the Mann-Whitney test. Time to event analysis is done using the Kaplan-Meier method. *P* values < 0.05 are considered statistically significant.

Data were analyzed using MedCalc© Statistical Software version 18.11.3 (MedCalc Software bvba, Ostend, Belgium; https://www.medcalc.org; 2019)

## Result

**Fig. 1:**
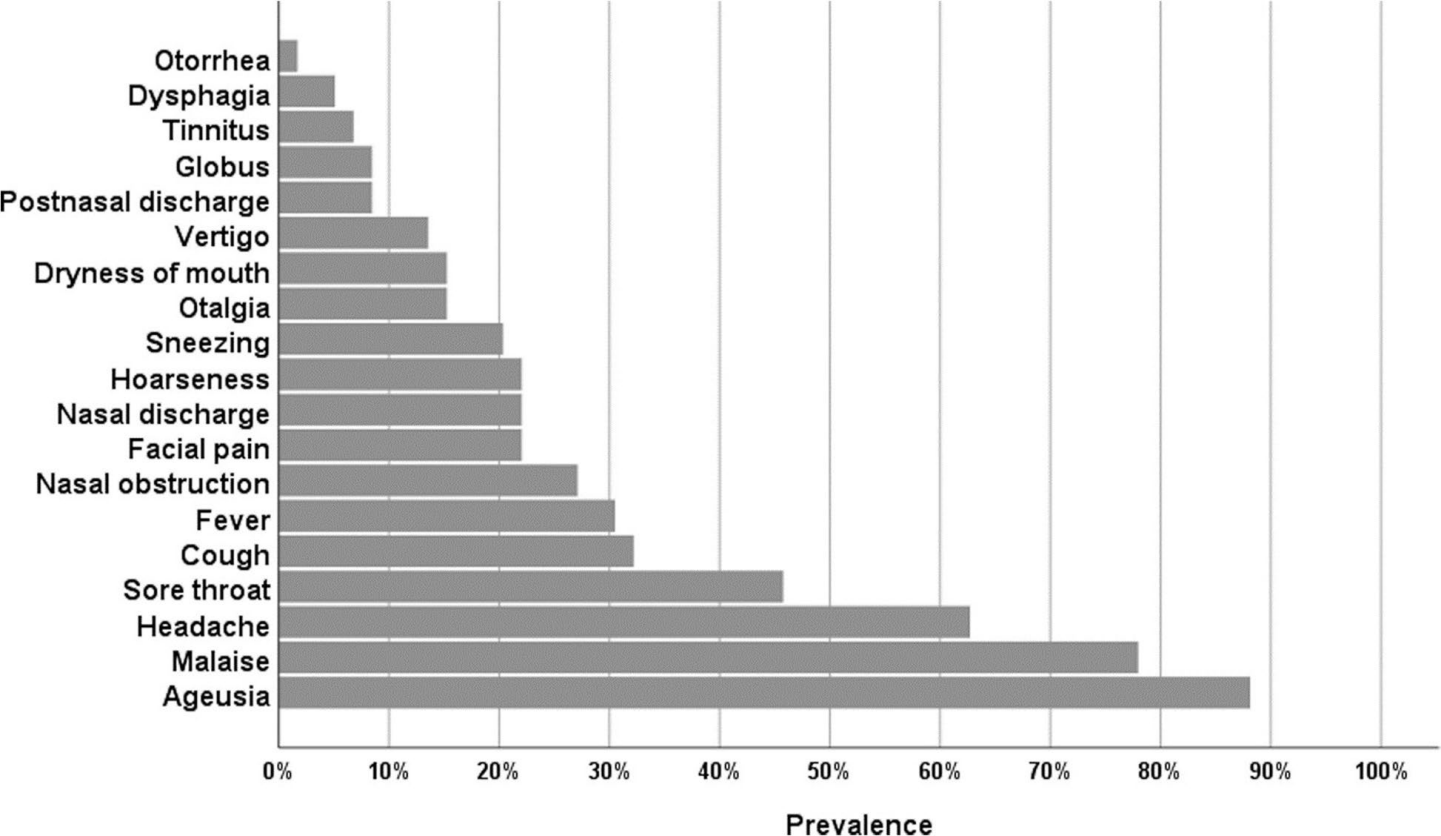
Prevalence of associated symptoms in the study population

Sixty patients fulfilled the inclusion and exclusion criteria, and were enrolled in the study. Their age ranged from 18 to 54 years with a mean age of 34.1 ± 8.4. The males were 33 (55%), while the females were 27 (45%). All patients have subjectively had an excellent smell and taste sensation before their loss or diminution of smell or taste. No history of recent travel was reported by all the study population. The majority of the cases were without a history of smoking (86%). The prevalence of the associated symptoms in the study population is shown in Fig. 1 and Table 1.

**Table 1:** Prevalence of associated symptoms in the study population

Symptom	Value
**General**	
Fever, *n* (%)	19 (31.7%)
Headache, *n* (%)	38 (**63**.3%)
Malaise, *n* (%)	47 (**78**.3%)
**Rhinological**	
Nasal obstruction, *n* (%)	16 (26.7%)
Nasal discharge, *n* (%)	13 (21.7%)
Post-nasal discharge, *n* (%)	5 (8.3%)
Sneezing, *n* (%)	12 (20.0%)
Facial pain, *n* (%)	13 (21.7%)
**Oropharyngeal**	
Ageusia, *n* (%)	52 (**88**.1%)
Dryness of mouth, *n* (%)	9 (15.0%)
Sore throat, *n* (%)	27 (**45**.0%)
Dysphagia, *n* (%)	3 (5.0%)
Globus, *n* (%)	5 (8.3%)
**Laryngeal**	
Hoarseness, *n* (%)	13 (21.7%)
Cough, *n* (%)	19 (**31**.7%)
**Otological**	
Otalgia, *n* (%)	9 (15.0%)
Otorrhea, *n* (%)	1 (1.7%)
Tinnitus, *n* (%)	4 (6.7%)
Vertigo, *n* (%)	8 (13.3%)

*n* number

The most common associated symptom was ageusia 52/60 (88.1%), followed by malaise 47/60 (78.3%), headache 38/60 (63.3%), and sore throat 27/60 (45.0%). Other symptoms compromise less than 40% (Table 1).

**Fig. 2:**
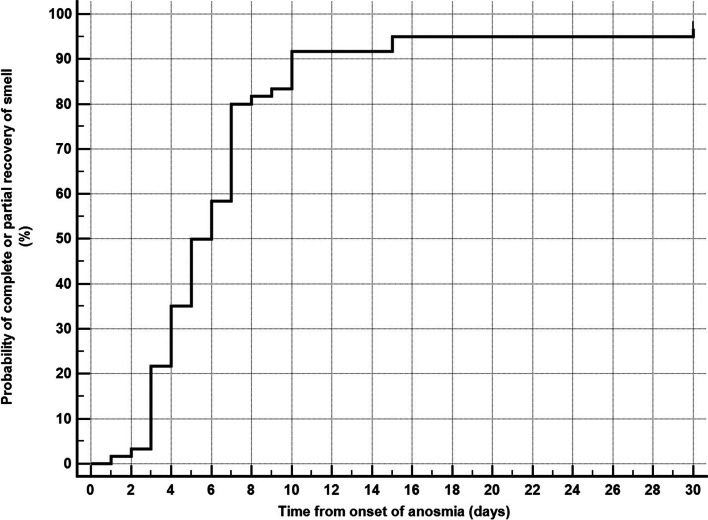
Kaplan-Meier curve for time to complete or partial recovery of smell. Median time to complete or partial recovery = 5 days (95% CI = 5 to 7 days)

**Fig. 3:**
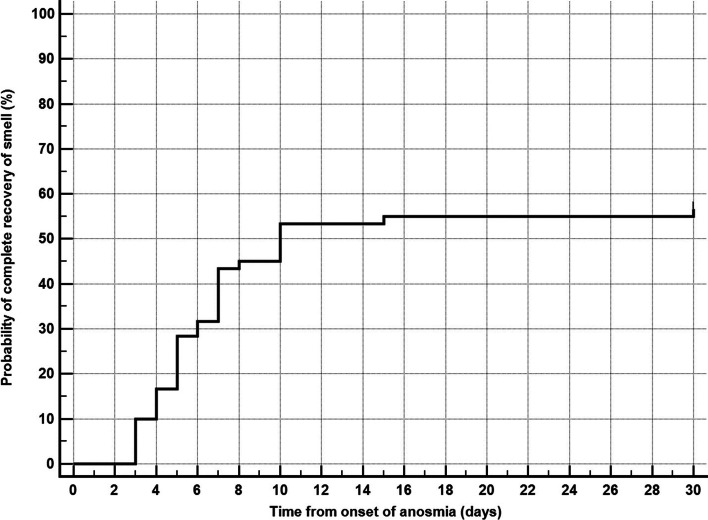
Kaplan-Meier curve for time to complete recovery of smell. Median time to complete recovery = 10 days (95% CI = 7 to 30 days)

Analysis of anosmia showed that 48 (80.0%) of patients had a sudden onset, while 12 (20.0%) had gradual onset. Complete anosmia and ageusia were observed in 51 (85%), and 9 (15.0%) had only hyposmia or hypogeusia. The interquartile range of anosmia duration was 5.5 days (4.0 to 7.0). Complete recovery of anosmia occurred in 34 patients (56.7%). Partial recovery in 24 (40.0%), and no recovery in 2 patients (3.3%) (Table 2). Kaplan-Meier curve showed that the median time to complete or partial recovery is 5 days (95% CI = 5 to 7 days) (Fig. 2), while the median time to complete recovery is 10 days (95% CI = 7 to 30 days) (Fig. 3).

**Table 2:** Characteristics of anosmia

Variable	Value
**Onset of anosmia**	
Sudden, *n* (%)	48 (80.0%)
Gradual, *n* (%)	12 (20.0%)
**Severity of anosmia**	
Complete, *n* (%)	51 (85.0%)
Partial, *n* (%)	9 (15.0%)
**Duration of anosmia (days), median (interquartile range)**	5.5 (4.0 to 7.0)
**Recovery of smell**	
No recovery, *n* (%)	2 (3.3%)
Partial recovery, *n* (%)	24 (40.0%)
Complete recovery, *n* (%)	34 (56.7%)

*n* number

Molecular testing by RT-PCR for COVID-19 was done through oropharyngeal and nasopharyngeal swabs for all patients. Forty-six (76.7%) were positive and 14 (23.3%) were negative. The mean cycle threshold at presentation (which indicates virulence of the virus) was 28.64 ± 6.40. After 1 month from the date of reporting smell loss, patients were subjected to a qualitative rapid test to detect IgM/IgG as shown in (Table 3). Negative samples for COVID-19 were tested by real-time multiplex PCR using FTD- respiratory 33 kit for the presence of a wide panel of respiratory pathogens (Table 4). Rhinovirus was the commonest isolated pathogen (5/14), followed by human parainfluenza (1/14), human adenovirus (1/14), and Enterovirus (1/14). Two bacterial pathogens were detected, while no pathogens could be detected in three patients.

**Table 3:** Results of laboratory workup

Variable	Value
**PCR at presentation**	
Negative, *n* (%)	14 (23.3%)
Positive, *n* (%)	46 (76.7%)
**Cycle threshold at presentation, mean ± SD (range**14 (23.3%)**)**	28.64 ± 6.40 (16.74 to 36.70)
**IgM after 1 month**	
Negative, *n* (%)	**31 (67.4%)**
Positive, *n* (%)	**15 (32.6%)**
**IgG after 1 month**	
Negative, *n* (%)	**20 (43.5%)**
Positive, *n* (%)	**26 (56.5%)**
**Patients with both IgG, IgM negative** *n* (%)	**13 (28.3%)**

*n* number

**Table 4:** Results of PCR testing for negative COVID-19 patients

Pathogen	Frequency
Rhinoviruses	5
Klebsilla pneumonia	2
Human parainfluenza	1
Staphylococcus aureus	1
Human adenovirus	1
Enterovirus	1
Negative for pathogen	3

**Fig. 4:**
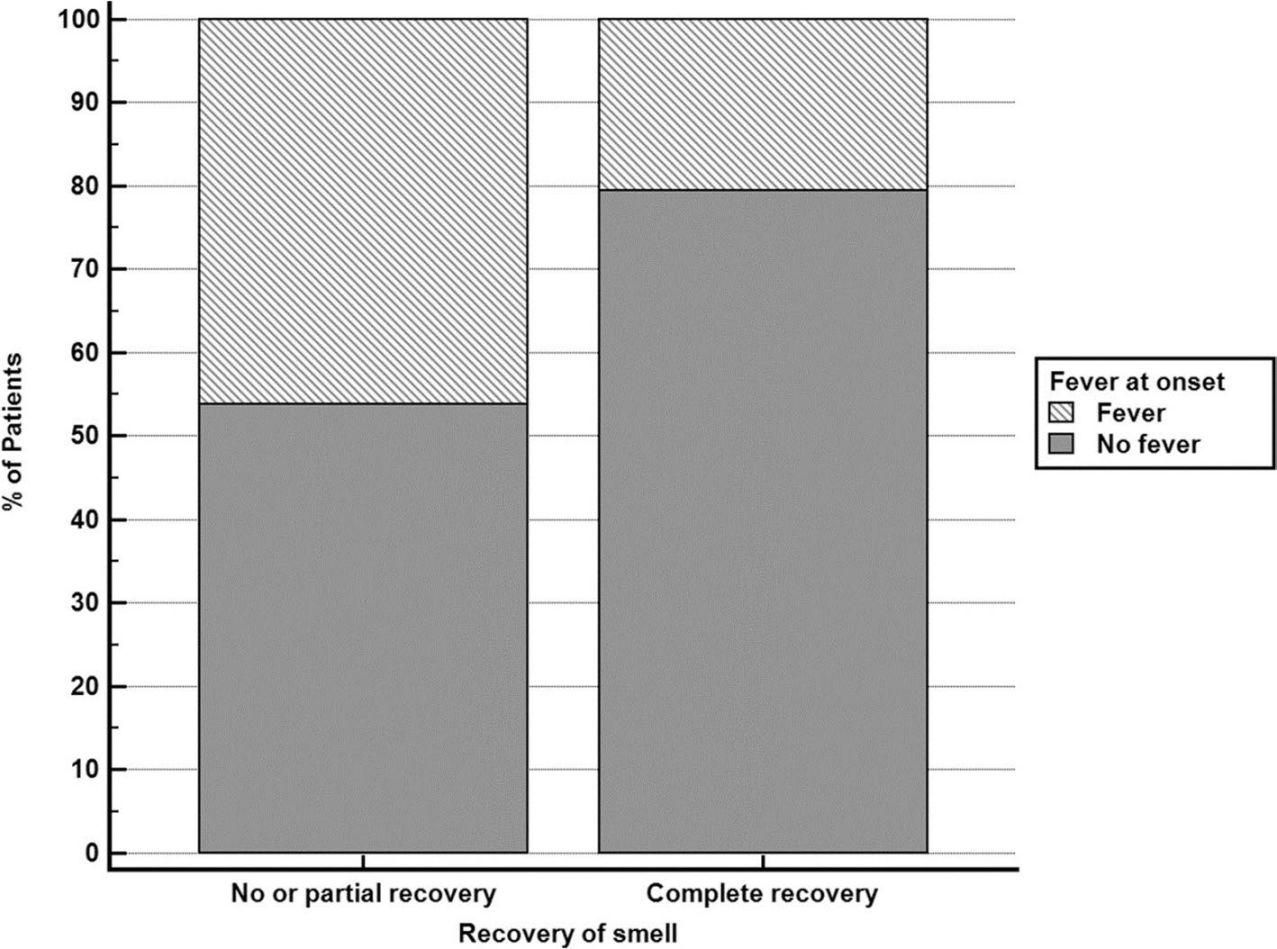
Relation between fever at onset and occurrence of complete recovery of smell. Fever is associated with lower probability of complete recovery (unadjusted odds ratio = 0.30, 95% CI = 0.10 to 0.94, *P* value = 0.035)

Statistical analysis of associations of recovery of smell with clinical and biochemical variables showed that only fever is associated with a lower probability of complete recovery (unadjusted odds ratio = 0.30, 95% CI = 0.10 to 0.94, *P* value = 0.035) (Fig. 4).

## Discussion

This study describes 60 patients who complained of anosmia, and 46 (76.7%) of them were confirmed to be COVID-19 positive. The symptoms of SARS-CoV-2 infection may range from an asymptomatic state, or symptomatic (e.g., fever, cough, headache, sore throat), up to acute respiratory distress syndrome [2, 11, 12] anosmia or ageusia might be the only symptom of the disease [13] or even it could be a sign of reinfection with COVID-19 [14].

The prevalence of anosmia varies between different studies; however, the pooled prevalence of anosmia within the infected COVID-19 population in a recent systematic review (including 32,142 COVID-19 patients) was approximately 38.2% [15]. Salmon et al. found that 94% of patients presenting to them with anosmia (a total of 55 patients) without nasal symptoms or upper respiratory symptoms tested positive for COVID-19 [16]. Our results showed that 76.7% were COVID-19-positive. Moreover, on removing patients who complained of nasal symptoms, the percentage is 77.5%, also on removing patients who complained of any other upper respiratory symptom, the percentage is 70%. The variation between the two studies might be due to ethnic variation, and more studies with larger numbers of patients are required to assess the incidence of COVID-19 infection within patients presenting with anosmia. On the other hand, our study supports Zayet et al. [17] who stated that anosmia has a **77**% positive predictive value for being COVID-19-positive.

Anosmia is not a new symptom in the otolaryngology practice, as trauma, viral infections other than SARS-CoV-2, neurodegenerative diseases were known causes for olfactory dysfunction [18]; hence, olfactory dysfunction is not only associated with SARS-CoV-2infection but also associated with different upper respiratory viral pathogens [1]. It was reported that anosmia was 10-fold higher with SARS-CoV-2 infection [19].

In the remaining COVID-19-negative 14 patients, we found that rhinoviruses were the most common viral pathogen detected in patients with anosmia. This finding is consistent with that of Suzuki et al. [1] who reported that rhinoviruses were detected in most of the patients who had post-viral olfactory anosmia. Those results reflect those reported previously, rhinoviruses were the most common respiratory pathogen that infected all age groups [20].

In our study, the most common associated symptom is ageusia (88.1%), followed by general symptoms: malaise (78.3%), headache (63.3%), then sore throat (45%), and cough (31.7%). Lechien et al *.*[21] also reported gustatory dysfunction was 89% in their study. Taste decreases with smell impairment regardless of the primary etiology [22]. Moreover, unlike other sensory modalities, taste and smell tend to show mutual fading, not compensatory mechanisms [23]. Headache and myalgia are the commonest general symptoms, while cough and sore throat were the commonest otolaryngologic manifestations associated with COVID-19 infection [2, 24–26]. No dyspnea was reported by any of our patients, as all of them were outpatients with the major complaint of anosmia without any respiratory distress.

Our study showed that 96.7% of the patients have recovered from anosmia (56.7% complete recovery, 40% partial recovery), while 3.3% (2 patients out of 60) showed no recovery at the end of the study follow-up (30 days); these results are in agreement with those obtained by Klopfenstein et al. [27] who reported that 98% of patients recovered within 28 days. We found the median time to complete recovery is 10 days (95% CI = 7 to 30 days). Also, it was found by Klopfenstein et al. [27] that the mean duration of anosmia was 8.9 days. It was suggested that recovery takes about 14 days, due to a reduction in viral load [28, 29]. By analyzing the association of recovery of smell with clinical and biochemical variables, we found that only fever is associated with a lower probability of complete recovery.

We detected the presence of antibodies against SARS-CoV-2 after 1 month from the onset of anosmia. We found that IgM was predominantly negative (67.4% of the patients), IgG was positive in 56.5% of the patients. Benazzo et al. [30] reported that the seroprevalence of SARS-CoV-2 IgG titer reached 80% after 3 weeks from the symptom onset. Also, it was found that IgG will remain in the serum for a longer duration. Hence, it may indicate a previous infection [31].

We found that 28.3% (13/46) of the patients showed negative antibody response for both IgG and IgM. In agreement with Liu et al. [32], they noted that consistently 34.3% of their patients were negative for IgM, and 14.3% were negative for all antibodies. So, mild cases may fail to show antibody response to SARS-CoV-2, and further studies are required to characterize the serological response of those cases of mild COVID-19 disease especially those who predominantly presented with anosmia as a major complaint. They reported also that the titers of severe cases for the total antibody were higher than those found in mild cases.

Our study has several limitations. First, we used SARS-CoV-2 RT-PCR using nasopharyngeal swabs which have a sensitivity of 56–83% [33] so one of the patients who tested negative may be infected with SARS-CoV-2 (false-negative), but we performed the RT-PCR panel of the upper respiratory viruses that came positive in 11 out of 14 negative patients. Second, objective assessment of smell or taste affection or recovery was not performed due to the high probability of being COVID-19 positive, but self-reporting is relatively accurate [34]. Especially, in case of interviewing the patient during the first visit, and after 1 month during the setting of performing the finger-prick test for the antibodies assessment. Third, we reported the outcome after one month so long-term follow-up is recommended. Fourth, we performed the antibody test only once after the infection instead of repeated measurements, and this was due to the limited availability of the test kits and also this was not the main objective of the study. Fifth, a limited number of patients enrolled in the study which was due to limited availability of resources, also anosmia being the only symptom that recovers within days was not a major motive for the affected patients to participate.

Variability among different studies may be due to dissimilarity in ethnicity, the number of studied populations, type of the population (age, hospitalized or not), method of assessment (subjective or objective), and the severity of the disease. To the best of our knowledge, this is the first study in North Africa that assessed the incidence of SARS-CoV-2 infection within patients with a major complaint of anosmia during the peak of the COVID-19 pandemic. We recommend further studies regarding smell dysfunction as a clinical indicator of COVID-19 infection especially when the prevalence of COVID-19 decreases.

## Conclusion

Sudden-onset anosmia is a symptom that is highly predictive of being COVID-19-infected. In a limited resource setting, in which testing for SARS-CoV-2 infection is not widely available, anosmia is a highly suspicious symptom that potentially necessitates self-isolation and may be used to screen asymptomatic carriers. COVID-19-related anosmia mostly recovers within 2 weeks.

## Data Availability

The datasets used during the current study are available from the corresponding author on reasonable request.

## Abbreviations

UK: United Kingdom
USA: United States of America
rt-PCR: Reverse transcriptase polymerase chain reaction
IgM: Immunoglobulin M
IgG: Immunoglobulin M
CI: Confidence interval
